# Universality and Uniqueness of Students’ Situational Interest in Physical Education: A Comparative Study

**DOI:** 10.5334/pb.446

**Published:** 2019-01-15

**Authors:** Cédric Roure, Vanessa Lentillon-Kaestner, Jacques Méard, Denis Pasco

**Affiliations:** 1 Université Catholique de Louvain, Louvain-la-Neuve, BE; 2 University of Teacher Education, Lausanne, CH; 3 University of Bourgogne Franche-Comté, Besançon, FR

**Keywords:** Motivation, Comparison, European countries, Secondary school

## Abstract

Situational interest (SI) has been conceptualized in physical education (PE) as a multidimensional construct including five dimensions: instant enjoyment, exploration intention, attention demand, novelty and challenge. Consistent with Ding, Sun and Chen (2013), who argued for the need ‘to develop cross-culture models to examine the universality of the motivation constructs’, the purpose of this study was to investigate the universality and uniqueness of students’ SI by comparing three French-speaking PE contexts. Participants were 1812 secondary school students from Belgium, France, and Switzerland. They responded to the French 15-item SI scale and the Total Interest scale (Roure, Pasco & Kermarrec, 2016) after practicing learning tasks in regular PE lessons. The relationships between the SI dimensions and total interest were compared between the three samples using correlation and regression analyses. A multivariate analysis of variance was also used to compare SI scores between the contexts. The results revealed that instant enjoyment and exploration intention were the two major motivating dimensions, highlighting the universality of students’ SI, whereas challenge and novelty revealed the uniqueness of this construct explored.

## Introduction

Researchers have demonstrated that students’ interest can be triggered, even if they have low self-efficacy, lack goals for learning, and are not able to self-regulate (Renninger & Hidi, 2011). A student’s choice to engage in a particular learning task reflects neither personality nor ability, but is dependent on the student’s perception of situational interest (Renninger & Hidi, 2016). Defined in the physical education (PE) context as ‘the appealing effect of the characteristics of an activity on an individual’ (Chen, Ennis, Martin & Sun, 2006: 3), situational interest (SI) has been used to interpret students’ motivation in task engagement. Accordingly, Chen, Chen and Zhu (2012) have found, in a meta-analysis related to the motivational constructs used to engage secondary school students in PE, that SI was a primary motivator for them.

The theory further postulates that SI is assumed to be transitory, environmentally activated, and context-specific (Renninger & Hidi, 2016). It is a kind of spontaneous interest that appears to fade as rapidly as it emerges, and is almost always content-specific (Renninger & Hidi, 2016). Its content specificity not only distinguishes SI from other motivational variables that focus on more general aspects of learning (e.g., achievement goals), but it also provides educators with information on how students’ motivation could be increased through SI development. According to Chen, Sun, Zhu and Chen (2014), SI has been conceptualized in PE as a multidimensional construct encompassing five dimensions: novelty, challenge, attention demand, exploration intention and instant enjoyment, and including an overall assessment of a task’s interest relating to a ‘total interest element’. Novelty relates to the difference between a student’s current knowledge and the knowledge which is required to learn in a task. Challenge refers to the difficulty of the task, as perceived by a student, in relation to his ability. Attention demand corresponds to a student’s cognitive involvement within a learning task. Exploration intention represents the characteristics of the learning tasks that encourage a student to discover and explore his environment. Instant enjoyment is defined as a positive feeling experienced by a student when participating in a learning task.

As SI is associated with variables that teachers have control over such as task design and teaching methods, it would be beneficial to the practice of PE if teachers understood how to use the SI dimensions to motivate students to learn (Ding, Sun & Chen, 2013; Hogheim & Reber, 2015; Patall, 2013). From a comparative perspective, the investigation of the SI dimensions and their relation to total interest in various PE contexts would help the understanding of the universality and uniqueness of students’ SI. When comparing the relationships between SI dimensions and those between the SI dimensions and total interest, high universality of students’ SI would correspond to a high level of similarities between the various PE contexts in terms of correlations and regressions from the SI dimensions toward total interest. On the contrary, when comparing the SI scores, high uniqueness would represent a high level of differences between the contexts in terms of mean differences related to the scores for the SI dimensions^1^. As SI is assumed to be content-dependent (Chen & Darst, 2001; Chen & Ennis, 2008), students’ SI should differ according to the characteristics of PE contexts. However, to date, only one study has made a comparison between PE contexts. Roure and Pasco (2018a) made a comparison between two PE contexts: the United States (US) and the French.

## Situational Interest in the French and US Physical Education Context

Based on Chen, Darst and Pangrazi’s (2001) previous study and a validated French SI scale (Roure, Pasco & Kermarrec, 2016), Roure and Pasco (2018a) investigated the relationships between SI dimensions and total interest in the French PE context. Results showed that instant enjoyment and exploration intention were the two major motivating dimensions that explained the variance in total interest. Consequently, Roure and Pasco (2018a) concluded that ‘teachers might design tasks that require not only physical engagement and enjoyment, but also higher-order cognitive processes demanding active exploration’ (p.16). For example, a learning task in badminton designed to promote attention demand and exploration intention could demand higher-order cognitive processes from students. In this task, two players play in a single game, where two lateral zones are identified along the length of the badminton court. The goal would be: the first player to win three points in these lateral zones using three different strikes (a smash, a drop shot, and a clear shot). This learning task provides: (1) attention demand through the need to keep track of the opponent’s position in relation to the lateral zones and shuttlecock trajectories, and (2) exploration intention through the choice of three different strikes to score points.

In the US context, Chen et al. (2001) had also mapped the relationships between the five SI dimensions and total interest with secondary school students. Their results revealed that instant enjoyment was a critical dimension for high SI, derived from the dimensions of exploration intention, attention demand and novelty. Accordingly, instant enjoyment was considered as ‘a process by itself during which a sense of becoming interested can be generated’ (Chen et al., 2001: 396). Although Roure and Pasco (2018a) clearly established differences between the American and the French contexts, some similarities have been observed in the relationships between the SI dimensions and total interest. In both contexts, instant enjoyment appeared to be a key dimension as well as exploration intention. Thus, it seems that these dimensions could be considered as reflecting the universality of students’ SI. Nevertheless, some differences remained due to the intrinsic nature of the contexts which are different in terms of aim, curriculum, content and assessment. For example, novelty had an indirect effect on total interest mediated by instant enjoyment in the US (Chen et al., 2001) whereas this dimension did not have any indirect effect on total interest in France (Roure & Pasco, 2018a). This could be explained as the National Association for Sport and Physical Education (NASPE) survey of 2001 in the US encouraged PE teachers ‘to teach a variety of physical activities that promoted PE classes as fun and enjoyable’ (Roure & Pasco, 2018a: 5). In French PE however, the novelty component had less effect on total interest, as this context is centered more on the teaching of the same physical activities throughout the curriculum in order to promote students’ cognitive development and learning methods (Roure, Kermarrec & Pasco, 2017). Given this, the novelty dimension seems to be related to the uniqueness of students’ SI.

Taking into account Roure and Pasco’s (2018a) results, a universalist approach to the study of SI dimensions was followed (Zusho & Clayton, 2011) in so far as it has the potential to reveal the universality and uniqueness of students’ SI. This approach is well-suited to compare various PE contexts since it posited ‘that there are certain basic psychological processes that are universal but also emphasizes the importance of culture and context’ (King & McInerney, 2014: 177).

## The Three French-speaking Contexts of PE

In this study, we focused on three different French-speaking contexts of PE (Wallonia (Belgium), the canton of Vaud (Switzerland), and France). These countries have a structured curriculum in PE, even though some differences are evident such as the curriculum aims and the physical activities used during the lessons. Because SI is content-dependent (Chen et al., 2001), the similarities and differences between these three contexts are important as they might be related to the universality and uniqueness of students’ SI.

In the French speaking community of Belgium (Wallonia), three groups of competencies were assigned to PE: the development of (a) physical fitness, (b) motor skills and (c) socio-motor coordination (Ministère de la Communauté Française [Ministry of French community], 2000). These aims are quite similar in the canton of Vaud in so far as PE pursues the development of motor skills, the acquisition of a healthy lifestyle and the development of self-awareness (Conférence intercantonale de l’instruction publique [Intercantonal conference of public instruction], (CIIP), 2010). In the same vein, the French PE context is characterized by the development of skills, competencies and knowledge related to physical activities, as well as the promotion of an active lifestyle (Ministère de l’Education Nationale [French Ministry of Education], (MEN), 2015). In terms of physical activities being taught in PE lessons, a balance between individual and collective, performance and personal-oriented and traditional and newly developed activities is encouraged in Belgium (Knop, Theeboom, Huts, De Martelaer & Cloes, 2005),^2^ whereas the French PE curriculum is defined by sports-centered activities with an emphasis on their cultural meaning and skills development (MEN, 2015). As a result, traditional and sports physical activities are taught, and students often experience the same activities since the teachers focus on understanding performance-related knowledge and ‘learning how to learn’ the PE content. The canton of Vaud, on the other hand, stands between the French and the Belgium PE contexts as they focus on traditional physical activities such as athletics, gymnastics and team sports. In contrast to the Walloon teachers who use an analytical approach for the teaching and learning processes, teachers in the canton of Vaud prioritize their relationships with their students, maintaining a sense of fairness between them (Allain, Deriaz, Voisard & Lentillon-Kaestner, 2015). Nevertheless, these two PE contexts try to enhance students’ levels of enjoyment within the lessons, which differentiate them from the French PE context which is more centered on learning processes and the students’ cognitive development (Roure et al., 2017).

## Purpose of the Study

Consistent with many researchers who have argued for the need to develop comparative studies to examine motivation constructs (Ding et al., 2013; King & McInerney, 2014; Zusho & Clayton, 2011), the purpose of this study was to investigate the universality and uniqueness of students’ SI by comparing three French-speaking PE contexts. From a comparative perspective, understanding the relationships between the SI dimensions and total interest in PE is significant. Firstly, this can lead to a deeper understanding of the SI construct in terms of universality and uniqueness. And secondly, the results of such study can inform teacher education programs about the SI dimensions most commonly perceived by students and consequently prepare preservice teachers better to meet, or enhance, current teaching practice standards. Referring to previous studies (Chen et al., 2001; Roure & Pasco, 2018a) and the comparison between the three French-speaking PE contexts, two assumptions were made. First, it was hypothesized that instant enjoyment and exploration intention would reflect better the universality of students’ SI, which means that these two dimensions would relate to total interest; all contexts tended to promote students’ enjoyment and encouraged them to discover new knowledge associated with the development of a healthy lifestyle. Second, it was expected that attention demand, novelty and challenge would reflect better the uniqueness of students’ SI, as the three contexts differed in terms of physical activities taught to students. Because these activities involved different competencies, knowledge and motor skills, differences between the scores for novelty and challenge were expected. Significant between-context differences in attention demand should also emerge depending on how the specific contexts promote students’ cognitive development.

## Method

### Participants

Participants were 1812 secondary school students from three French speaking countries: Belgium (the Walloon community), France (the Northwest region), and Switzerland (the canton of Vaud). Of the total number of participants, 735 were students in the Wallonia PE context (WALL-PE), aged between 12 and 18 years old (*M_age_* = 15.3, *SD* = 1.5), 49.2% boys, 601 were students in the French PE context (FRA-PE), aged between 11 and 18 years old (*M_age_* = 14.4, *SD* = 1.9), 51.4% boys, and 476 were students in the canton of Vaud PE context (VAUD-PE), aged between 12 and 17 years old (*M_age_* = 13.9, *SD* = 1.1), 54.2% boys. The ethical boards of the host universities gave permission to conduct the study and agreements were also obtained from the principals of the participating schools. The students’ parents were informed about the scope of the study and consent was requested from all of them. All parents allowed their children to participate in the study.

### Measures

#### Situational interest

The French 15-item SI scale (Roure et al., 2016) was used to measure students’ SI during the learning tasks. The scale includes five SI dimensions: novelty (e.g., ‘what we did today was new to me’), instant enjoyment (e.g., ‘what we did was enjoyable for me’), exploration intention (e.g., ‘I wanted to analyze and have a better handle on what we were learning today’), attention demand (e.g., ‘what we were learning demanded my high attention’), and challenge (e.g., ‘what we were learning was hard for me to do’). Each dimension of SI consists of three items. The items were arranged randomly and each was rated on a five-point Likert scale ranging from 1 = *strongly disagree* to 5 = *strongly agree*.

#### Total interest

The French 4-item scale (Roure et al., 2016) was used to measure students’ total interest during the PE lesson. The scale is unidimensional and consists of four items (e.g, ‘what we were learning was interesting for me to do’). The items were arranged randomly and each was rated on a five-point Likert scale ranging from 1 = *strongly disagree* to 5 = *strongly agree*.

### Data Collection

In line with Matsumoto and Hee Yoo (2006: 243) who argued that ‘the most important concept that researchers need to be aware of when conducting cross-cultural research is equivalence’, equivalence was addressed through the collection of data. The teachers involved in this study were told to teach their lessons as usual. They were male and female, full-time certified PE teachers with teaching experience ranging from eight to 25 years. Due to the large sample size of participants and also to the three different PE contexts, data were collected on a variety of physical activities taught in the PE lessons. These physical activities were dependent on the students’ classes and countries: FRA-PE (Athletics, Badminton, Baseball, Basketball, Climbing, Dance, Gymnastics, Handball, Kayaking, Orienteering, Swimming, Volleyball and Wrestling), WALL-PE (Badminton, Basketball, Climbing, Gymnastics, Handball, Hockey, Kinball, Volleyball and Ultimate frisbee), and VAUD-PE (Athletics, Basketball, Dance and Gymnastics). Depending on each PE context, students participated in a physical activity unit consisting of four to eight lessons. For the purpose of the study, the lesson situated at the middle of the unit for all physical activities was chosen for the data collection. Immediately after completing a learning task set by the teachers in this lesson, students responded to the French SI scale (Roure et al., 2016). They also filled in the Total Interest scale (Roure et al., 2016) at the end of the same lesson. The data were collected by the researchers under the supervision of the students’ own PE teacher. To minimise students’ tendency to give socially desirable responses, students were encouraged to answer honestly and assured that their responses would remain anonymous and confidential.

### Data Analyses

Students’ responses were aggregated respectively to total interest and to the five dimensions of SI. After conducting preliminary analyses, data were analysed following two stages. Firstly, based on the taxonomy of possible cross-cultural differences established by King and McInerney (2014), confirmatory factor analyses (CFA) were conducted to verify the structure of the SI construct in each sample and to test for configural and metric equivalence between the samples. Maximum likelihood estimation was used to evaluate the fit of the CFA measurement models to the empirical data. Acceptable model fit was assessed using multiple indices commonly used: RMSEA, CFI, TLI and NFI (Blunch, 2008; Hu & Bentler, 1999). The second stage consisted of examining the differential salience, in so far as ‘some psychological factors may be more relevant or more salient in one context compared to another’ (King & McInerney, 2014: 182). Correlations and multiple regressions were used and compared between the three samples, to analyze the relationships between the SI dimensions and total interest. Additionally, a multivariate analysis of variance (MANOVA) was performed to analyze the statistical significance of the sample differences in the dimensions of SI and total interest. Version 23.0 of SPSS (SPSS Inc, Chicago, IL) and version 22.0 of AMOS software were used for all statistical analyses.

## Results

### Preliminary Analyses

Analysis of the skewness and kurtosis values revealed that the data were normally distributed and no problem of multicollinearity between variables was found. Internal consistencies of the scales were good with Cronbach’s alphas of .82 for total interest in FRA-PE (.83 in WALL-PE and .84 in VAUD-PE), .82 for instant enjoyment in FRA-PE (.81 in WALL-PE and .82 in VAUD-PE), .81 for exploration intention in FRA-PE (.77 in WALL-PE and .78 in VAUD-PE), .84 for attention demand in FRA-PE (.85 in WALL-PE and .78 in VAUD-PE), .79 for challenge in FRA-PE (.85 in WALL-PE and .71 in VAUD-PE) and .88 for novelty in FRA-PE (.93 in WALL-PE and .88 in VAUD-PE), respectively. Due to the hierarchical nature of the data (i.e. students data nested in classes and in schools), the amount of variance explained by school-level and class-level variance was analyzed. Results for the school-level variance showed that the intraclass correlation (ICC) for students’ total interest ranged between .039 (for VAUD-PE) and .066 (for WALL-PE), meaning that between-schools variability accounted for a maximum of 6.6% of the variance of students’ total interest. Similarly, the ICC for the five SI dimensions ranged between .011 (for exploration intention in FRA-PE) and .058 (for instant enjoyment in WALL-PE), indicating a low between-schools variance. Results for the class-level variance were quite similar with ICC ranging between .012 (for attention demand in VAUD-PE) and .087 (for challenge in WALL-PE). Under those circumstances and according to Preacher, Zhang and Zyphur (2011), multilevel analysis would have been less efficient as ICCs are lower than .10 for all study variables. Therefore, we proceeded with student-level analysis.

### Differential Factor Structures

The measurement model of all five latent constructs and 15 indicators (for the five SI dimensions) yielded good fit to the data in each sample: FRA-PE, *χ*^2^ (115) = 317.39; *χ*^2^/*df* = 2.76; CFI = .97; NFI = .95; TLI = .95; RMSEA = .054 with CI_90_ = .047–.061; WALL-PE, *χ*^2^ (132) = 346.19; *χ*^2^/*df* = 2.62; CFI = .97; NFI = .96; TLI = .97; RMSEA = .047 with CI_90_ = .041–.053; and VAUD-PE, *χ*^2^ (133) = 337.42; *χ*^2^/*df* = 2.54; CFI = .95; NFI = .93; TLI = .93; RMSEA = .057 with CI_90_ = .049–.064. The factor loadings of the indicators ranged between .67 and .89 for FRA-PE, .71 and .93 for WALL-PE, and .63 and .89 for VAUD-PE, indicating good construct validity in all three samples. Comparison of the measurement model with a model where all factors loadings of the variables were constrained showed a Δ*χ*^2^ (21) = 28.76, *p* < .11, ΔCFI < .01, guaranteeing configural and metric equivalence between the three samples. In the same vein, the measurement model of the latent construct and four indicators (for total interest) yielded good fit to the data in each sample: FRA-PE, *χ*^2^ (30) = 54.49; *χ*^2^/*df* = 1.82; CFI = .98; NFI = .97; TLI = .97; RMSEA = .034 with CI_90_ = .027–.041; WALL-PE, *χ*^2^ (32) = 60.33; *χ*^2^/*df* = 1.88; CFI = .97; NFI = .96; TLI = .97; RMSEA = .041 with CI_90_ = .036–.048; and VAUD-PE, *χ*^2^ (33) = 59.21; *χ*^2^/*df* = 1.79; CFI = .98; NFI = .97; TLI = .97; RMSEA = .033 with CI_90_ = .025–.039. The factor loadings of the indicators ranged between .76 and .89 for FRA-PE, .75 and .91 for WALL-PE, and .77 and .92 for VAUD-PE, indicating good construct validity in all three samples. Comparison of the measurement model with a model where all factors loadings of the variables were constrained showed a Δ*χ*^2^ (4) = 6.28, *p* < .09, ΔCFI < .01, guaranteeing configural and metric equivalence between the three samples. Therefore, the comparison of the structural effects was legitimate, which allowed the differential salience to be analyzed.

### Differential Salience

Means, standard deviations, and correlations among the study variables are presented in Table 1 for all contexts. The results revealed similarities and differences between the three samples. In terms of similarities, instant enjoyment and exploration intention were strongly and positively related to total interest, with correlation coefficients ranging between .57 and .78. In addition, challenge was correlated positively to attention demand and novelty with coefficients ranging between .23 and .54. Finally, exploration intention correlated positively to attention demand and instant enjoyment with coefficients ranging between .31 and .57. Differences were associated with challenge as this variable was correlated positively with total interest (*r* = .13, *p* < .01) in FRA-PE whereas it correlated negatively with total interest (*r* = –.09, *p* < .01) in WALL-PE, and did not correlate with this variable in VAUD-PE. Additionally, challenge was only correlated negatively with instant enjoyment in WALL-PE (*r* = –.26, *p* < .01). Finally, challenge correlated positively with exploration intention in the three samples but with differences considering their respective coefficients (*r* = .31 for FRA-PE, *r* = .23 for WALL-PE, *r* = .14 for VAUD-PE, *p* < .01).

**Table 1:** Descriptive statistics and correlations between study variables in the three contexts.

	Context	Min	Max	Mean	*SD*	1		2		3		4		5		6

1. Total interest	FRA	4	20	12.94	3.77	–										
	WALL	4	20	12.95	3.70	–										
	VAUD	4	20	12.15	4.13	–										

2. Challenge	FRA	3	15	7.68	3.10	.13	*	–								
	WALL	3	15	7.48	3.40	–.09	*	–								
	VAUD	3	15	10.45	2.86	–.02		–								

3. Attention demand	FRA	3	15	10.11	3.12	.28	*	.48	*	–						
	WALL	3	15	9.32	3.26	.12	*	.54	*	–						
	VAUD	3	15	9.40	2.91	.28	*	.53	*	–						

4. Instant enjoyment	FRA	3	15	9.91	3.24	.78	*	–.02		.20	*	–				
	WALL	3	15	10.94	2.89	.74	*	–.26	*	.02		–				
	VAUD	3	15	8.56	3.28	.78	*	–.08		.19	*	–				

5. Novelty	FRA	3	15	6.80	4.07	.14	*	.38	*	.36	*	.11	*	–		
	WALL	3	15	8.13	4.78	.01		.23	*	.14	*	.01		–		
	VAUD	3	15	11.23	3.91	–.01		.39	*	.23	*	–.04		–		

6. Exploration intention	FRA	3	15	8.41	3.13	.63	*	.31	*	.41	*	.51	*	.26	*	–
	WALL	3	15	8.17	3.09	.57	*	.23	*	.36	*	.35	*	.11	*	–
	VAUD	3	15	10.11	3.20	.70	*	.14	*	.31	*	.57	*	.12	*	–

*Note*: FRA: French PE context; WALL: Wallonia PE context; VAUD: Vaud PE context; * *p* < .01.

As in the procedures used by Chen et al. (2001) and Roure and Pasco (2018a), a series of multiple-regression analyses were conducted to examine whether SI dimensions could predict total interest. In FRA-PE, the main predictors of total interest were instant enjoyment (*β* = .63, *p* < .01) and exploration intention (*β* = .30, *p* < .01), accounting for 69% of its variance. Similarly, instant enjoyment (*β* = .61, *p* < .01) and exploration intention (*β* = .37, *p* < .01) were positive significant predictors of total interest in WALL-PE, accounting for 66% of its variance. These two SI dimensions were also positive predictors of total interest in VAUD-PE (*β* = .54 for instant enjoyment and *β* = .38 for exploration intention, *p* < .01), but were associated with two other predictors, namely attention demand (*β* = .11, *p* < .01) and challenge (*β* = –.08, *p* < .01). These four predictors explained 72% of the variance of total interest. All multiple-regression analyses are presented in Table 2.

**Table 2:** Series of multiple-regression analyses.

	French PE context				Wallonia PE context				Canton of Vaud PE context			

Predictor	*β*		Adjust *R*^2^		*β*		Adjust *R*^2^		*β*		Adjust *R*^2^	

Total interest			.69	**			.66	**			.72	**
Challenge	.05				–.00				–.08	**		
Attention demand	.02				–.02				.11	**		
Instant enjoyment	.63	**			.61	**			.54	**		
Novelty	–.03				–.03				–.02			
Exploration intention	.30	**			.37	**			.38	**		
Instant enjoyment			.29	**			.24	**			.36	**
Challenge	–.24	**			–.41	**			–.21	**		
Attention demand	.08	*			.09	*			.14	*		
Novelty	.04				.04				–.06			
Exploration intention	.54	**			.40	**			.56	**		
Exploration intention			.39	**			.27	**			.38	**
Challenge	.20	**			.18	**			.07			
Attention demand	.20	**			.25	**			.15	**		
Novelty	.05				.03				.08	*		
Instant enjoyment	.46	**			.39	**			.55	**		

*Note*: *β*: Standardized beta coefficient; t: test value; * *p* < .05, ** *p* < .01.

As multiple correlations were demonstrated between the SI dimensions, we tested the multiple-regression analyses with the predictors of total interest as dependent variables (i.e., instant enjoyment and exploration intention) and the other SI dimensions as independent variables. Results showed that for the three samples, exploration intention positively predicted instant enjoyment whereas challenge was a negative significant predictor with coefficients ranging between .40 and .56 for the former and between –.21 and –.41 for the latter. Additionally, the main predictors for exploration intention were instant enjoyment (with coefficients ranging between .39 and .55) and attention demand (with coefficients ranging between .15 and .25) for all samples. Differences were observed for challenge, in so far as it was a positive predictor for exploration intention in FRA-PE and WALL-PE, and for novelty which positively predicted exploration intention in VAUD-PE.

The results from MANOVA revealed a significant main effect in SI and total interest scores for the different samples, Pillaï Trace = .41, *F*(6,1804) = 77.60, *p* < .001, *η*^2^ = .21. Table 3 reports the means, standard deviations and differences between the samples based on SI measures.

**Table 3:** Differences between situational interest measures across the three PE contexts.

	French PE context		Wallonia PE context		Canton of Vaud PE context		*F*(2, 1809)	η^2^

	*M*	*SD*	*M*	*SD*	*M*	*SD*		

Total interest	12.94^a^	3.77	12.95^a^	3.70	12.15^b^	4.12	7.46*	.01
Instant enjoyment	9.92^a^	3.24	10.94^b^	2.89	8.56^c^	3.28	84.77*	.09
Exploration intention	8.41^a^	3.13	8.17^a^	3.09	10.11^b^	3.20	61.09*	.06
Attention demand	10.11^a^	3.12	9.32^b^	3.26	9.40^b^	2.92	11.84*	.01
Novelty	6.80^a^	4.07	8.13^b^	4.78	11.23^c^	3.91	143.31*	.14
Challenge	7.68^a^	3.10	7.48^a^	3.40	10.45^b^	2.86	145.38*	.14

*Note*: F: test value; * *p* < .001; η^2^: effect size. ^a, b, c^: these values are significantly different from each other.

Follow-up ANOVAs revealed slight differences in the total interest and attention demand scores: VAUD-PE reported lower scores for total interest whereas FRA-PE received higher scores for attention demand. Small effect sizes were observed for the differences between the samples on exploration intention and instant enjoyment. VAUD-PE reported higher scores for exploration intention. Higher scores were observed for instant enjoyment in WALL-PE compared to FRA-PE which also reported higher scores than VAUD-PE. Finally, moderate effect sizes were found for novelty and challenge. VAUD-PE reported higher scores for challenge. Higher scores were observed for novelty in VAUD-PE compared to WALL-PE which also reported higher scores than FRA-PE.

## Discussion

The purpose of this study was to investigate the universality and uniqueness of students’ SI by comparing three French-speaking PE contexts. In line with Zusho and Clayton (2011), we adopted a universalist approach to the study of SI as it is the most appropriate way of looking at these three contexts from a motivational science perspective. This approach holds ‘that there are certain basic psychological processes that are universal but also emphasizes the importance of culture and context’ (King & McInerney, 2014: 177). Therefore, the results are discussed regarding both the universality and uniqueness of students’ SI in relation to the two assumptions made.

### Universality of Students’ SI

Testing for differential factor structures, this study demonstrates configural and metric equivalence between the samples, indicating that the SI construct is valid in all three contexts. Furthermore, differential salience investigation also reveals similarities between the three contexts, highlighting the universal aspects of SI. With respect to our first assumption, our data revealed that instant enjoyment and exploration intention reflect better the universality of students’ SI. These two dimensions of SI are the major positive predictors in regression analyses of total interest in all three contexts. This is congruent with many studies which have revealed strong connections between enjoyment, interest and exploration of information (Ainley & Ainley, 2011; Chen et al., 2001; Rotgans & Schmidt, 2011). For instance, in the context of science education, Ainley and Ainley (2011) demonstrated positive relationships between interest, the desire to find out more about a specific topic and feelings of enjoyment. Similarly, Chen et al. (2001) highlighted in their study that ‘rather than merely providing a variety of different and new physical activities to students, physical education teachers should use exploration-oriented learning tasks to enhance students’ feeling of instant enjoyment and situational interest in learning those activities’ (p. 397). All things considered, by revealing that instant enjoyment and exploration intention have a strong impact on students’ total interest in each of the three present contexts, this study is congruent with research literature. Studies have demonstrated that higher-order cognitive processes demanding active exploration and enjoyment represent key factors underlying the motivation for students to maintain a positive engagement in PE (Jaakkola, Wang, Soini, & Liukkonen, 2015; Roure & Pasco, 2018b).

By revealing that challenge is the third universal aspect of SI, the results slightly invalidate the first assumption as challenge was not expected to reflect the universality of students’ SI. However, the challenge component is not a major contributor for the universal aspects of SI as the three contexts only shared the negative relationship with challenge toward instant enjoyment (with coefficients ranging between –.21 to –.41). Because challenge is defined as the level of difficulty relative to one’s ability (Chen et al., 2014), students’ perception of ability can explain the negative relationship between challenge and instant enjoyment. As students experience enjoyment and motivation when a task they are involved in is comparable to their level of perceived ability (Fairclough, 2003), this negative relationship can be interpreted as the need to find an optimal challenge in learning tasks. Consequently, PE teachers might create learning tasks that are optimally challenging to maintain students’ perceived ability and increase their instant enjoyment.

### Uniqueness of Students’ SI

Our second assumption pertained to the uniqueness of students’ SI. Our results revealed that the mean scores of the SI dimensions and total interest significantly differed among the three samples, highlighting the unique aspects of SI. According to this result, the second assumption is partially validated as differences were observed beyond the dimensions of attention demand, novelty and challenge. However, except for the attention demand component, challenge and novelty were the two dimensions in which the differences among the samples were most apparent (with the highest effect size, η^2^ = .14). With respect to the dimension of challenge, the mean score for this dimension was higher in VAUD-PE. This particularity of VAUD-PE could be attributed to the fact that teachers do not prioritize students’ performance or motor skills (Allain et al., 2015) and thus are not primarily centered on students’ ability levels when designing learning tasks. Another interpretation might be that there is a relationship between challenge and novelty as students perceived high levels of challenge and novelty when practicing the learning tasks. The novelty component of the content could lead students to perceive learning tasks as more challenging. Since the level of challenge seems to exceed students’ perceived ability, this interpretation is congruent with Ding et al. (2013) who also found that a high level of challenge did not positively contribute to SI when their students reported high levels of novelty. The relation between challenge and novelty could also explain why low scores for these two dimensions tend to coincide in FRA-PE and WALL-PE. Because the French students often experience the same activities to develop and understand performance-related knowledge, it is plausible that they perceived the learning tasks as usual and not particularly challenging. Theses results are more surprising in WALL-PE in so far as a balance between traditional and newly developed activities is encouraged (Knop, Theeboom, Huts, De Martelaer & Cloes, 2005). However, the analytical approach for the teaching and learning processes, which is often used by teachers, may tend to prioritize the traditional activities and thus reduce the perception of novelty. Moreover, under this approach, the content taught to students is divided into smaller units as progressive steps to reach a more complex goal. These smaller units can be perceived as easier by students. All in all, this should remind teachers to build optimally challenging tasks with a mix of novel and familiar elements in order to increase students’ SI (Roure & Pasco, 2018a).

Besides the relation between challenge and novelty, the novelty component clearly highlights the uniqueness of students’ SI as it is associated with major differences across the samples (with the highest effect size, η^2^ = .14). In comparison to the other contexts, the students in VAUD-PE reported the highest score for novelty. According to Allain et al. (2015), teachers often change the physical activities they teach students, depending on the pupil-teacher relationships, negotiating with their students to choose the physical activities and learning tasks. This emphasis on novelty is well founded to promote students’ SI since Shen, Winger, Li, Sun and Rukavina (2010) revealed that students demonstrate a lack of interest when learning tasks are repetitive in nature. Furthermore, the highest score for novelty in VAUD-PE is associated with the highest score for exploration intention. This relation is relevant since providing the students with the opportunity to make choices in the learning environment has repeatedly been associated with SI and intrinsic motivation (Hogheim & Reber, 2015). In contrast to the VAUD-PE context, French students reported the lowest score for novelty. This result seems logical considering that teachers in FRA-PE are encouraged to frequently use the same physical activities throughout the curriculum. Learning tasks are also performed many times by students to improve their skills and knowledge, as PE is focused on ‘developing performance proficiency in skills, understanding performance-related knowledge and learning how to learn the PE content’ (Roure & Pasco, 2018a: 6).

Even if instant enjoyment and exploration intention reflect the universality of students’ SI, it appears that these two dimensions also play a role in the unique aspects of SI. However, it should not be considered that the second assumption is invalidated since the effect sizes explaining the differences between the three contexts for these two dimensions are lower than those for challenge and novelty. Thus, exploration intention is the third component that contributes to the uniqueness of students’ SI, especially in VAUD-PE where students reported the highest score for this dimension. In conjunction with the high scores for novelty and challenge in VAUD-PE, the relationships between these three SI dimensions need to be interpreted. Since novelty is a positive correlate of exploration intention and that challenge can lead to an exploration of the environment possibilities, students’ exploration intention is promoted. This interpretation is congruent with Shen (2017) who wrote: ‘When designed to provide differentiated success (e.g., choices of high, medium, and low difficulty levels within the same task), exploratory tasks arouse learners’ perceptions of situational interest, increasing their intrinsic motivation to engage in the activity’ (p. 614). By enhancing novelty within the PE content, Swiss teachers encouraged their students to discover a greater range of physical activities. ‘When teachers offer students activity, partner or equipment choices within differentiated tasks, they strengthen situational interest and facilitate interest internalization found in learning tasks’ (Shen, 2017: 614).

Finally, instant enjoyment represents the last SI dimension which highlights the unique aspects of this construct. FRA-PE is characterized by the highest score for instant enjoyment, which is congruent with findings from Benhaim-Grosse (2007) indicating that almost three-quarters of the French students reported that PE provided them with enjoyment and positive feelings. As enjoyment is seen as the most important affective consequence of quality PE (Cairney et al., 2012), the results from WALL-PE are not surprising and are in line with the promotion of students’ enjoyment observed in classes (Knop et al., 2005). The lower score for instant enjoyment in VAUD-PE seems rather unexpected, especially as Swiss teachers consider their relationships with students as a major concern (Allain et al., 2015). An interpretation can be made referring to the conceptualization of enjoyment in PE (Hashim, Grove and Whipp, 2008). According to these authors, Swiss teachers could be focused on the enjoyment derived from ‘teacher-generated excitement’ as they prioritize their relationships with students and not on the enjoyment derived from ‘activity-generated excitement’. Since they primarily focus on their relationships with students rather than on the development of motor skills (Allain et al., 2015), there is a possibility that students do not perceive a high level of instant enjoyment, as this dimension is content-dependent (Chen et al., 2001; Roure & Pasco, 2018a).

### Conclusions, Limitations and Future Directions

In conclusion, the universalist approach adopted in this study (Zusho & Clayton, 2011) acknowledges that there seems to be good evidence to support the universality of students’ SI across different French-speaking PE contexts. However, it was also revealed that the scores for the SI dimensions were different between the three samples, indicating that the contexts indeed played a major role. Therefore, the uniqueness of students’ SI in PE is also important, which is congruent with the content-dependence of this construct (Chen et al., 2001; Chen & Ennis, 2008). According to the two assumptions made for this study, it could be noted that some SI dimensions reflect the universality of this construct better (i.e., instant enjoyment and exploration intention) whereas others demonstrate its uniqueness better (i.e., novelty and challenge).

Because it has been demonstrated that SI is associated with variables that teachers have control over such as: task design and teaching methods (Hogheim & Reber, 2015; Patall, 2013; Roure & Pasco, 2018b), it would be interesting to present some practical implications for PE teachers in order to enhance students’ SI. In line with previous suggestions made by Roure and Pasco (2018a), PE teachers might create learning tasks that stimulate students’ enjoyment and their willingness to explore their environment. By eliciting various positive emotional processes from students, high levels of students’ enjoyment can be reached. Moreover, students’ exploration intention can be increased by involving them in decision making, which will give them the freedom to make choices and the opportunity to affect the way learning tasks are carried out. Simultaneously, to enhance the enjoyment and exploration intention components, teachers need to pay attention to students’ perceived ability of a presented task. To reach an optimal challenge for most of their students, teachers should differentiate the learning tasks by providing multi-levels of difficulties and several possibilities for the students’ progression. Finally, PE teachers should encourage students’ attention demand by creating cognitive tasks and by enhancing students’ mental representations, which in turn could facilitate their willingness to explore the different aspects of their environment.

Three limitations of the study should be considered when interpreting or generalising our findings. Firstly, the present study included cross-sectional data across three different PE contexts. Future studies could collect longitudinal data to analyse how SI might change over time, which would inform on the design of practical interventions. Secondly, it should be noted that our samples are not nationally representative but were collected in specific regions of France, Belgium and Switzerland. Consequently, generalization of the present results would require additional samples from these countries. Thirdly, even though this study shows the universality and uniqueness of students’ SI, it cannot ‘pinpoint the cultural ingredient responsible for cross-cultural variations’ (King & McInerney, 2014: 189). Further investigations, conducted as experimental approaches, are needed to examine the effects of cultural and contextual factors on students’ SI.
